# Accurate Measurement of Canal Length during Root Canal Treatment: An *In Vivo* Study

**Published:** 2015-03

**Authors:** Durre Sadaf, Muhammad Zubair Ahmad

**Affiliations:** College of Dentistry, Qassim University, Saudi Arabia

**Keywords:** Electronic Apex Locator (EAL), Apex locators malfunction, Electronic working length determination, In Vivo

## Abstract

**Objectives:**

To assess the consistency and accuracy of Electronic Apex Locator (EAL) (Root ZXII) in individual canals and its association with other clinical variables.

**Study Design:**

Cross-Sectional study.

**Place of study:**

Dental section of the Aga Khan University Hospital, Karachi, Pakistan.

**Materials and Methods:**

Working length was measured by EAL in 180 patients requiring endodontic therapy in molar and premolar teeth. The effects of clinical variables e.g. gender and pulpal status on the consistency and accuracy of EAL were recorded. Performance of apex locator was considered “Consistent” when the scale bar was stable and moved only in correspondence to the movement of file in the root canal. Accuracy was determined by inserting the file at the working length determined by the EAL and periapical view of radiograph was taken using paralleling technique. Estimated working length was considered accurate when the file tip was located 0-2mm short of the radiographic apex. If the file was overextended from the radiographic apex, it showed dysfunction of the EAL.

**Results:**

Consistency of EAL was found 97.6% in distobuccal canals, 91.1% in palatal canals, 73.7% in mesiolingual canals, 83.3% in mesiobuccal and 80.2% in distal canals. Accuracy of EAL was 91.4% in mesiolingual canal, 92% in mesiobuccal, and 90.2% in Palatal and 93.2% in distal canal.

**Conclusion:**

Consistency of electronic apex locator vary in different canals, however consistent measurements are highly accurate. No significant association was found between other clinical variables with the consistency and accuracy of EAL.

## INTRODUCTION

Accurate determination of working length is one of the most important steps in endodontic therapy. Inaccurate determination of working length may lead to short or overextended obturation. Short working length may result in retained necrotic tissues in apical area and overextended working length may result in over-instrumentation and over-obturation. According to American Association of Endodontics 2003, working length is defined as ‘the distance from a coronal reference point to the point at which the canal preparation and filling should terminate (1). Minor diameter or apical constriction is the place where instrumentation and obturation should terminate. Sometimes, it may coincide with the cementoenamel junction, where transition of the pulpal tissues with the periodontal tissues takes place (2). Radiographs, Electronic apex locators and operator’s tactile sensation are methods used for determination of working length. Apical constriction is located 0.5-0.75 mm coronal to the Major foramen which in turn is located 0.5mm coronal to the apical terminus (3, 4). Working length by radiographs is measured 0.5mm-1.0mm short of the radiographic apex of the tooth. However, radiographic method has been found to be associated with shortening or elongation, interpretation variability and lack of three dimensional representations (5). Working length 1mm short of the radiographic apex is not always reliable and may result in over or under instrumentation (5, 6). Working length determination by electronic method was first done by Custer in 1918 (7). Suzuki in 1942 developed first electronic apex locator (8). This device was resistance based and measured the resistance between two electrodes. Later devices were impedance-based (9).

“The consistency of a device describes the regularity of its function. A measuring device that is able to give a reading each time used is considered to function consistently regardless of the quality of the performance. The quality of the performance/measurements can be described in terms of reliability which is the probability that a device will perform a required function with high accuracy, repeatability and reproducibility” (10). Electronic apex locators are considered highly reliable and superior to radiographic methods in terms of accuracy and consistency (10-14). However electronic apex locators are also not without flaws. Inconsistent readings or no readings signify dysfunction of electronic apex locators (10). There are very few studies on inconsistent functioning of EALs (10). It is recommended to use radiographs with electronic apex locators for verification. The reasons for inaccuracy of electronic apex locators are still not clear.

Most of the studies are done to assess the accuracy of EAL (15-17). In previous study (10), the tooth was taken as a unit of analysis while in this study individual root canals were analyzed for working length by Root ZXII (J. Morita Corp., Tokyo, Japan).

Objectives of this study are to assess dysfunction (inconsistent measurements) of electronic apex locator Root ZX II in individual canals and the association of consistency and accuracy of electronic apex locator with clinical factors such as gender, type of canal and pulp vitality that may affect the consistency and accuracy of electronic apex locators.

## MATERIALS AND METHODS

Clearance from Ethical Review Committee of University was obtained. One hundred and eighty patients (115 females and 65 males) requiring endodontic treatment were selected from outpatient dental section, the Aga Khan University Hospital, Karachi Pakistan. Maxillary and mandibular molars and premolars were included. The study was performed by a single operator. The age of the patients was between 12-70 years. Teeth with immature apices, root resorption and metallic restorations were excluded.

**Figure 1:**
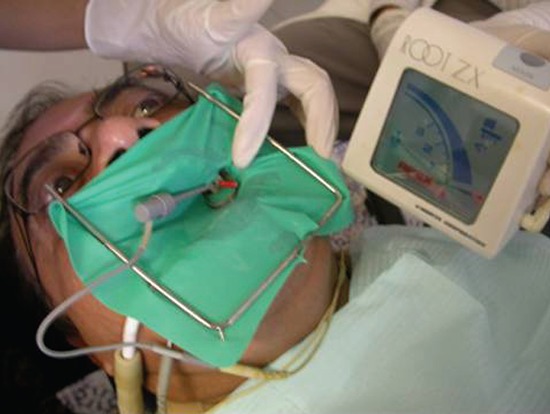
Electronic Apex Locator Root ZX II in use. Note that bar is stable and its position is indicating the position of endodontic file in the root canal space.

Preoperative working length was measured on radiograph. Rubber dam was applied. Endodontic access preparation was done. Patency of the canal was assessed with # K-10 or # K-15. The root canals were flared in coronal and middle third by Gate-Glidden burs G1, G2 and G3 followed by S1 and S2 ProTaper files. Root canals were irrigated by 5.25% sodium hypochlorite. An appropriate hand file that was largest and reached to the estimated working length was selected. The consistent and inconsistent function of EAL was recorded in a similar manner as described by Ashraf (10). The function of the apex locator was recorded to be “consistent” when the scale bar of the apex locator was stable and only moved in correspondences to the movement of the file. Its function was considered “inconsistence” when the displayed bar intermittently flashed, rapidly moved from one position to another and when no bar displayed (Fig. 1).

**Figure 2:**
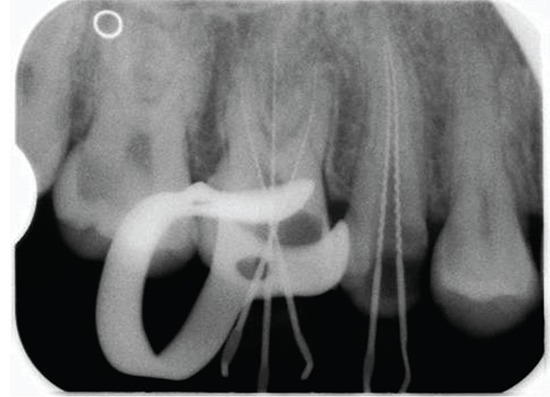
Endodontic files within 2 mm of the radiographic apex of teeth.

The selected file was advanced in the root canal until it reached green bar. The stopper of the file was adjusted and periapical view of radiograph using parallel technique was taken. If working length was within 2mm of the radiographic apex, it was considered acceptable and denotes the accuracy of the Root ZX II (J. Morita Corp., Tokyo, Japan) (Fig. 2). If the file tip extended beyond the apex, it was “overextended” and reflects dysfunction of the EAL. If the file tip was more than 2mm short of the apex, it was considered “short”. Clinical parameters like age and sex of the patient, preoperative pain, preoperative tenderness to percussion and endodontic diagnosis (Acute pulpitis, chronic pulpitis, and apical periodontitis) were recorded.

## RESULTS

Frequency and distribution of gender, type of teeth, preoperative pain, preoperative tenderness to percussion (TTP), diagnosis is presented in Table 1. Radiographic lengths were compared with Consistency of EAL in the root canals using χ^2^ test (Table 2).

**Table 1:** Frequency distribution of gender, type of teeth, diagnosis, preoperative pain and Preoperative Tenderness to Percussion (TTP)

Gender Distribution	Male	36.1%
	Female	63.9%
Type of Teeth	MaxillaryMolars	24.4%
	Mandibular Molars	53.9%
	Maxillary Premolars	8.9%
	Mandibular Premolars	12.8%
Diagnosis	Apical Periodontitis	48.3%
	Acute Pulpitis	35.0%
	Chronic Pulpitis	16.7%
Preoperative Pain	Symptomatic	58.9%
	Asymptomatic	41.1%
Preoperative TTP	Positive	27.2%
	Negative	72.8%

**Table 2:** Consistency and Accuracy of Electronic Apex Locator (EAL) in different canals

Canals	Consistency of EAL	Accuracy	Inconsistency of EAL	Accuracy	*P* Value (Chi Square test)

MB	83.3%	92%	16.7%	0.0%	<0.001
ML	73.7%	91.4%	26.3%	4.0%	<0.001
DB	97.6%	90.2%	2.4%	100%	0.905
P	91.1%	90.2%	8.9%	40%	<0.001
D	80.2%	93.2%	19.8%	0.0%	<0.001

MB, Mesiobuccal canal; ML, Mesiolingual canal; DB, Distobuccal canal; P, palatal canal; D, distal canal.

EAL showed consistent readings in 83.3% of Mesiobuccal (MB) canals. These readings were accurate in 92% of cases. EAL showed inconsistent readings in 16.7% of MB canals. None of these readings were accurate. The association between consistency and accuracy was statistically significant (*P* value <0.001) (Table 2).

In cases of consistent function of EAL in MB canals the working length file was overextended in 0.8% of cases and it was short in 7.2% of cases (Table 3).

**Table 3:** Frequency of overextended and short working length files in cases of consistent and inconsistent readings of EAL

Root Canal	Consistency of EAL	Overextended working length files	Short working length files	Inconsistency of EAL	Overextended working length files	Short working length files

MB	83.3%	0.8%	7.2%	16.7%	87.5%	66.7%
ML	73.3%	1.4%	7.1%	26.3%	20%	76%
DB	97.6%	0.0%	9.8%	2.4%	0.0%	0.0%
P	91.1%	2.0%	7.8%	8.9%	60%	0.0%
D	80.2%	2.7%	4.1%	19.8%	38.9%	61.1%

MB, Mesiobuccal canal; ML, Mesiolingual canal; DB, Distobuccal canal; P, palatal canal; D, distal canal.

In cases of inconsistent function of EAL in MB canals the working length file was overextended in 87.5% of cases and it was short in 66.7% of cases (Table 3).

EAL showed consistent readings in 73.3% of Mesiolingual (ML) canals. These readings were accurate in 91.4% of cases. EAL showed inconsistent readings in 26.3% of MB canals. These readings were accurate in 4.0% of cases. The association between consistency and accuracy was statistically significant (*P* value 0.001) (Table 2).

In cases of consistent function of EAL in ML canals the working length file was overextended in 1.4% of cases and it was short in 7.1% of cases. (Table 3).

 In cases of inconsistent function of EAL in ML canals the working length file was overextended in 20% of cases and it was short in 76% of cases. (Table 3).

EAL showed consistent readings in 97.6% of Distobuccal (DB) canals. These readings were accurate in 90.2% of cases. EAL showed inconsistent readings in 2.4% of MB canals. 100% of these readings were accurate. The association between consistency and accuracy was significant statistically (*P* value <0.001) (Table 2).

In cases of consistent function of EAL in DB canals the working length file was overextended in none of cases and it was short in 9.8% of cases (Table 3).

In cases of inconsistent function of EAL in DB canals the working length file was overextended in none of the cases and it was short in none of the cases (Table 3).

EAL showed consistent readings in 91.1% of palatal canals. These readings were accurate in 90.2% of cases. EAL showed inconsistent readings in 8.9% of palatal canals. These readings were accurate in 40% of cases. The association between consistency and accuracy was statistically significant (*P* value <0.001) (Table 2).

In cases of consistent function of EAL in palatal canals the working length file was overextended in 2.0% of cases and it was short in 7.8% of cases (Table 3).

In cases of inconsistent function of EAL in palatal canals the working length file was overextended in 60% of cases and it was short in none of the cases (Table 3).

EAL showed consistent readings in 80.2% of distal canals. These readings were accurate in 93.2% of cases. EAL showed inconsistent readings in 19.8% of distal canals. None of these readings were accurate. The association between consistency and accuracy was statistically significant (*P* value <0.001) (Table 2).

In cases of consistent function of EAL in distal canals the working length file was overextended in 2.7% of cases and it was short in 4.1% of cases (Table 3).

In cases of inconsistent function of EAL in distal canals the working length file was overextended in 38.9% of cases and it was short in 61.1% of cases (Table 3).

Association of consistency of EAL with preoperative tenderness to percussion was not significant (*P*=0.690). Association of consistency of EAL with pulpal status such as acute pulpitis, chronic pulpitis and apical periodontitis was not significant (*P*=0.655). Association of consistency of EAL with preoperative pain was not significant (*P*=0.5).

Association of gender with consistency was not found significant (*P*=0.660).

## DISCUSSION

Accuracy of EALs are well documented, however there are very few studies on consistency of the EALs (10). Consistency was related to the movement of scale bar on the electronic apex locator moving along with the file as reported by ElAyouti (10). Accuracy of electronic apex locators was assessed with the help of radiographs. If radiographic length of the file was within 2mm the apex, it was considered accurate.

The Root ZX II (J. Morita Corp., Tokyo, Japan) is a third generation EAL. It uses multiple frequencies and can be used in the presence of different electrolytes in the canal.

Root ZXII (J. Morita Corp., Tokyo, Japan) has been found superior to radiographs in its ability to locate apical constriction (5). Accuracy of Root ZXII is superior to other electronic apex locators (10).

According to Clayton (18), Radiographs are not reliable tool for working length determination. He stated that when a file appears long on radiograph, it is actually longer by 1.2 mm. ElAyouti reported that electronic apex locator reduced the frequency of overestimation of working length. (19).

Kobayashi and Suda (20) described that the Root-ZX measures impedance values at two frequencies (8 kHz and 0.4 kHz) and calculates a quotient of impedances. This quotient is expressed as a position of the file in the canal. When the minor diameter of the canal is reached, the quotient approaches a value of 0.67. This is a constant value that is reliable in the presence of electrolytes or pulp tissue.

In this study Root canals were preflared with Gate-Glidden Burs #1-3 before determination of working length with Root ZX II (J. Morita Corp., Tokyo, Japan). Preflaring has been found to significantly increase the accuracy of EALs (21).

Consistency of Root ZX II(J. Morita Corp., Tokyo, Japan) is seen from 73%- 97% in this study. According to ElAyouti, dysfunction or inconsistency of Root ZX II was seen in every sixth patient (10). In his study, consistency was found from 85%-97% (10). Dunlap found accuracy in Root ZX model about 82% (22). In another in vivo study, the accuracy of Root ZX is 88.9% (23).

Variations in consistent measurements of EAL in different canals may be due to variations in canal curvature, canal width, and internal conditions of the canal such as presence of tissues and fluids. Ebrahim and Yoshioka reported that diameter and size of the file may affect the consistent function of the Root ZX (24).

Canal curvature was found to effect on function of apex locators. Apex locators are capable of assessing apical constriction in straight and wider canals with greater accuracy as compared to narrow and curved canals.

Hoer noted that EALs which are based on impedance quotient methods are only 57% successful in accurate determination of apical constriction (25).

Consistent measurements were found 90% accurate. ElAyouti found accuracy about 97% (10). Inconsistency of EAL was more associated with overextended radiographic length than short radiographic length.

Short measurement is not always inaccurate as apical constriction sometimes may be found 3 mm short of apex (25). Thus accuracy of the EAL may be much higher than that we found in this study as our accuracy of EAL is only limited to the measurement within 2 mm of the apex. In contrast the long measurements were definitely unacceptable because over instrumentation took place. Preoperative tenderness to percussion has not been found associated with consistency of the EAL. Gender, pulpal status, preoperative symptoms have been found no association with consistency of electronic apex locators. However in teeth presented with preoperative symptoms, greater inconsistency of EAL was found as compared to asymptomatic teeth. This finding was similar to ElAyouti (10) who described inconsistent reading of EAL in teeth with irreversible pulpitis.

## CONCLUSION

Consistency of electronic apex locator vary from 73.3%-97.6% in different canals, however consistent measurements are highly accurate, found between 90.2%-93.2%. Canal Curvature seemed to affect the consistency of EAL. Consistent readings of EAL were seen in straight canal (Palatal) more commonly than curved canals (MB, ML). Inconsistent measurements are highly associated with overextended working length. Symptomatic teeth gave more inconsistent reading to EAL than asymptomatic teeth.

Electronic apex locators are highly consistent and accurate devices in determining working length of root canals in endodontic therapy. However, role of radiographs cannot be completely eliminated in working length determination it is advised to use this devices in conjunction with radiographs.
